# Supplementary material for: DNA Methylation as a Biomarker for Monitoring Disease Outcome in Patients with Hypovitaminosis and Neurological Disorders
Source: Genes (Basel). 2023 Jan 30;14(2):365. doi: 10.3390/genes14020365 (PMC9956161; doi:10.3390/genes14020365)
Supplement: Supplementary file 1 [file genes-14-00365-s001.zip › genes-2124655-supplementary.pptx]

## Slide 1
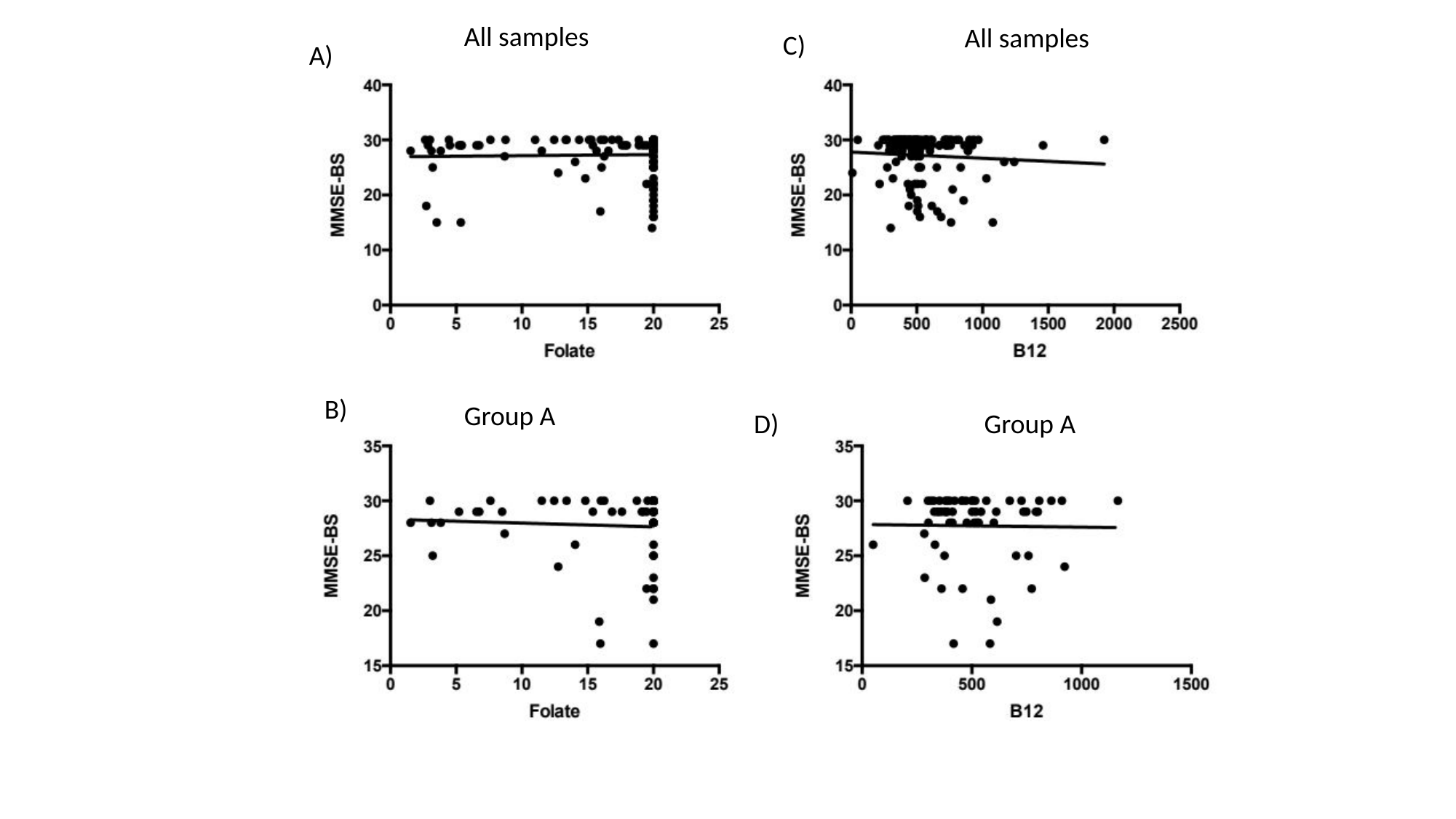

All samples
All samples
C)
A)
B)
Group A
D)
Group A

## Slide 2
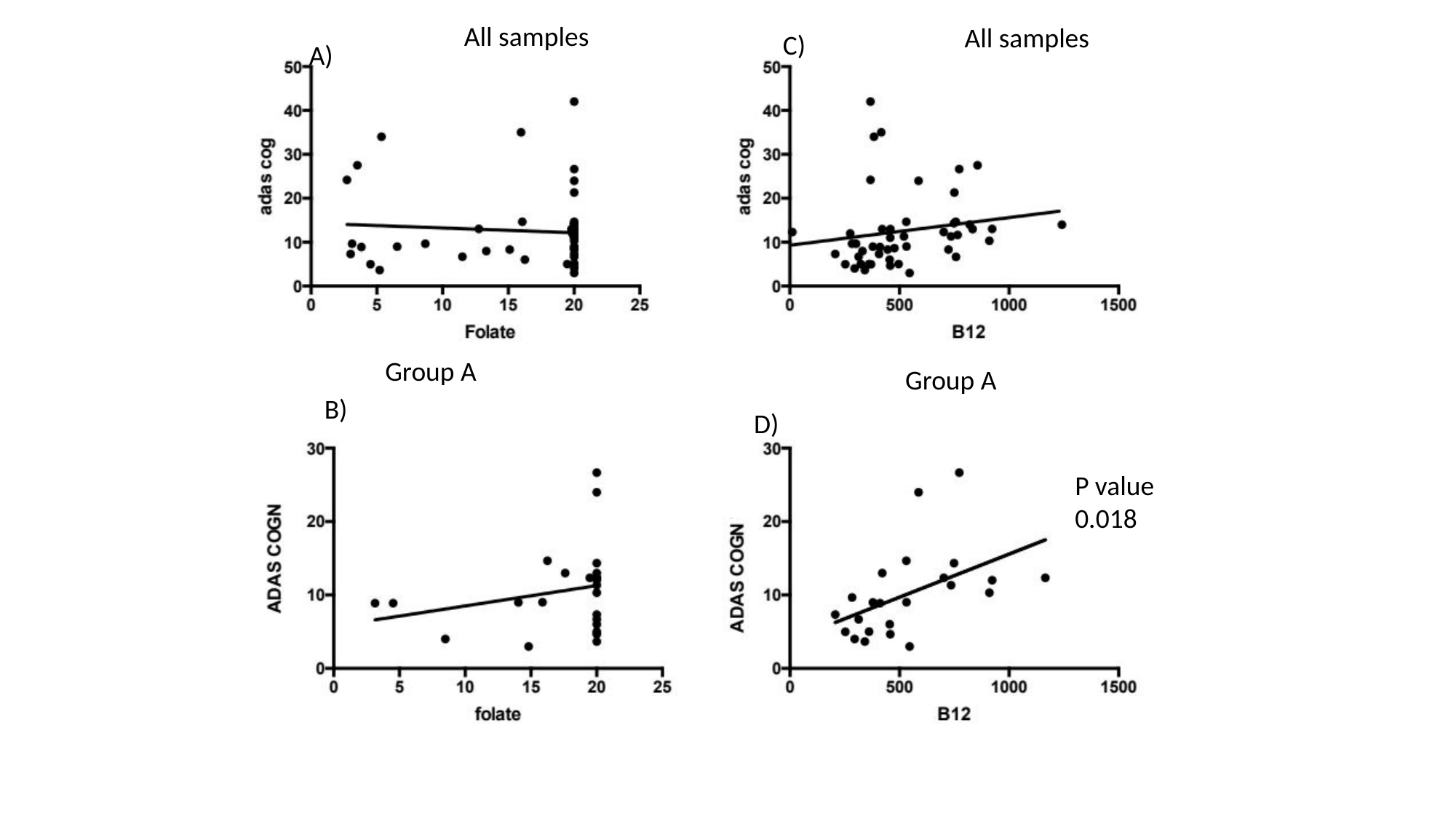

All samples
All samples
C)
A)
Group A
Group A
B)
D)
P value 0.018
